# Supplementary material for: Mutual reinforcement of land-based carbon dioxide removal and international emissions trading in deep decarbonization scenarios
Source: Nat Commun. 2024 Aug 21;15:7160. doi: 10.1038/s41467-024-49502-8 (PMC11339383; doi:10.1038/s41467-024-49502-8)
Supplement: Supplementary file 1 — Supplementary Information [file 41467_2024_49502_MOESM1_ESM.pdf]

Supplementary Information for:

Mutual Reinforcement of Land-based Carbon Dioxide Removal and International Emissions Trading in Deep Decarbonization Scenarios

Jennifer Morris[1*], Angelo Gurgel[1], Bryan Mignone[2], Haroon Kheshgi[3] and Sergey Paltsev[1]

[1] Massachusetts Institute of Technology, Cambridge, MA, USA

[2] ExxonMobil Technology and Engineering Company, Annandale, NJ, USA

[3] University of Illinois at Urbana-Champaign, Urbana, IL, USA

[*]Corresponding Author: holak@mit.edu

1. Additional Figures for Main 2°C Scenarios

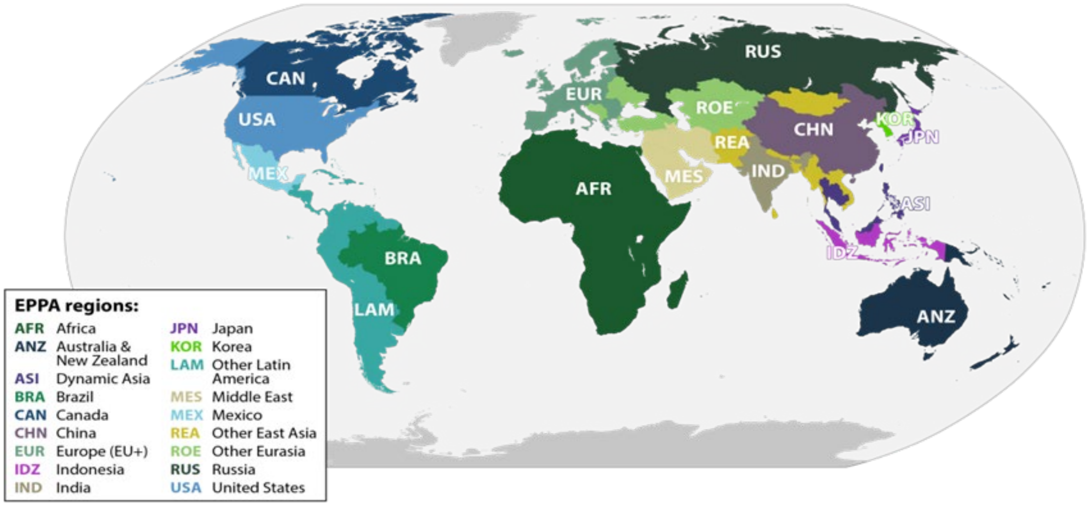

Supplementary Figure 1. **Regional representation in the Economic Projection and Policy Analysis (EPPA) model.** These regions are further aggregated into two regional groupings: Developed and Emerging economies. The Developed economies regional grouping includes Australia & New Zealand (ANZ), Dynamic Asia (ASI), Canada (CAN), Europe (EU+, EUR), Japan (JPN) Korea (KOR) and the United States (USA). The Emerging economies regional grouping includes Africa (AFR), Brazil (BRA), China (CHN), Indonesia (IDZ), India (IND), Other Latin America (LAM), Middle East (MES), Mexico (MEX), Other East Asia (REA), Other Eurasia (ROE), and Russia (RUS).

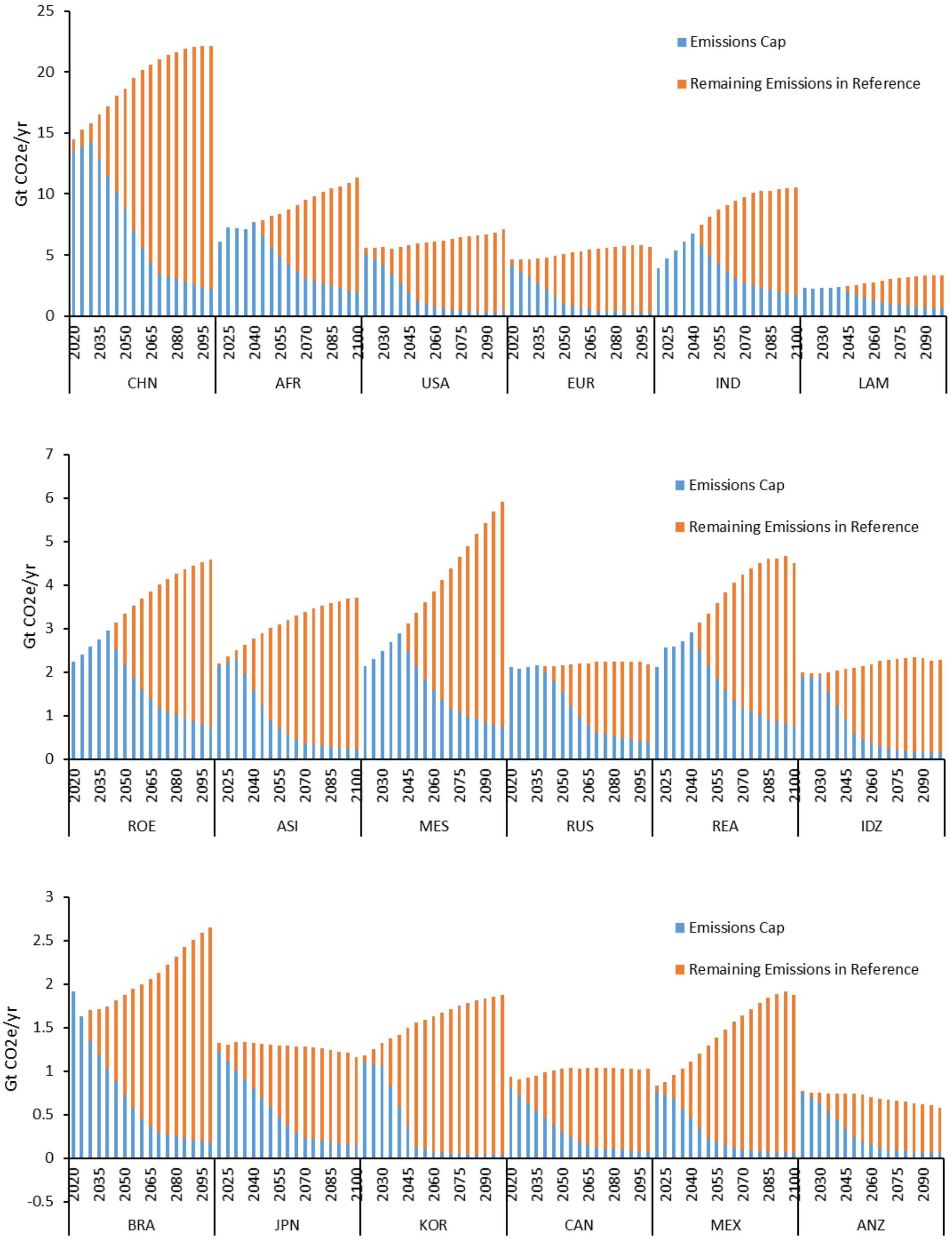

Supplementary Figure 2. **Regional greenhouse gas (GHG) emissions caps.** The GHG emissions caps to achieve the 2°C stabilization target are shown in blue, and the remaining (uncapped) emissions in the reference scenario are shown in orange. Note the different scales for each panel.

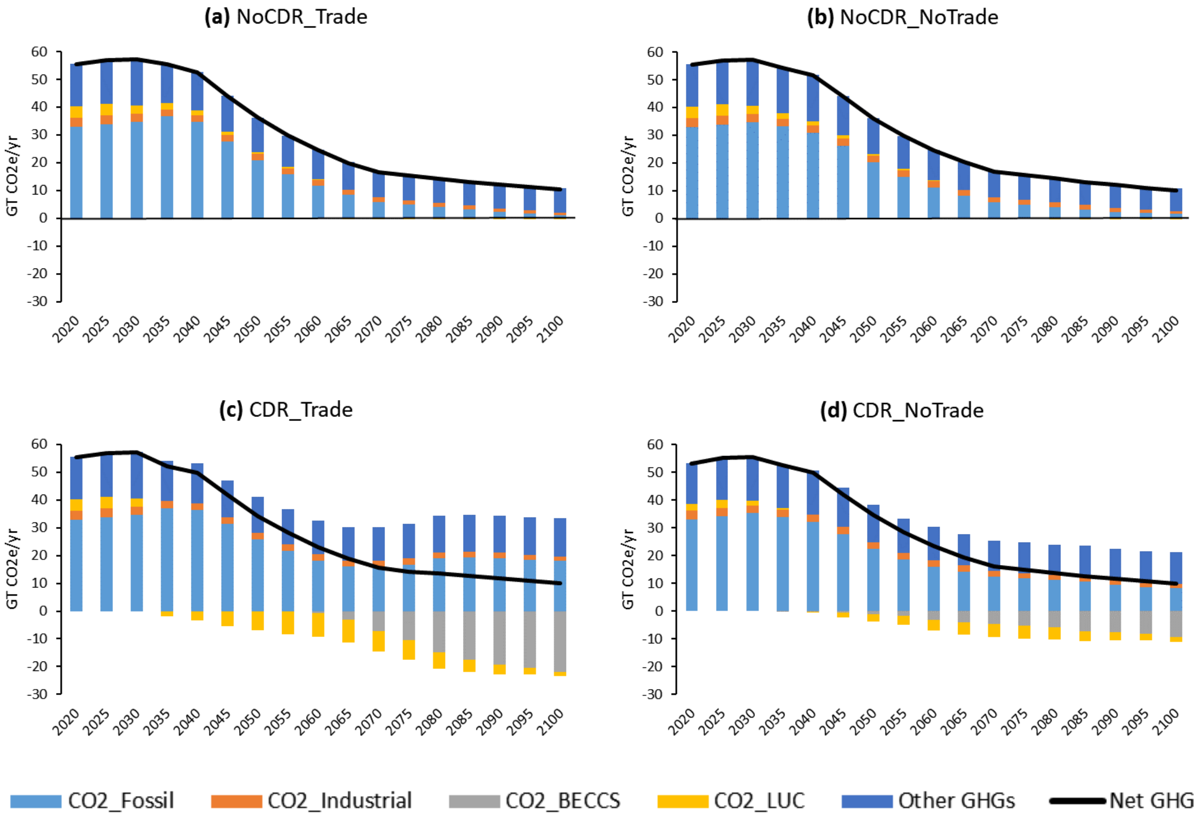

Supplementary Figure 3. Global Greenhouse Gas (GHG) emissions by type in four main 2°C scenarios. **(a)** NoCDR_Trade, **(b)** NoCDR_NoTrade, **(c)** CDR_Trade, and **(d)** CDR_NoTrade.

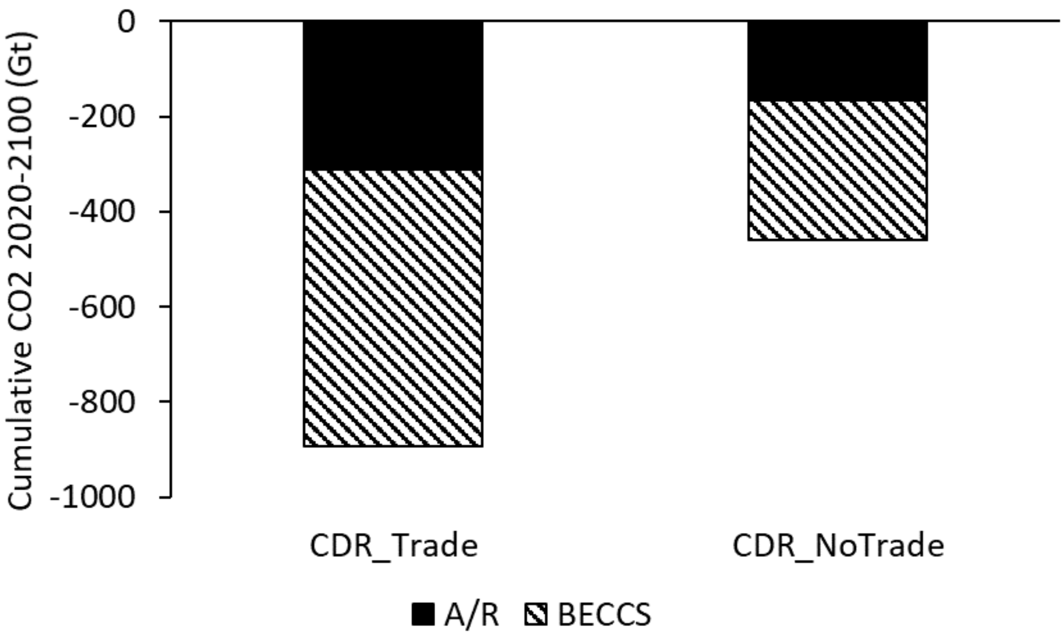

Supplementary Figure 4. **Global cumulative carbon dioxide removal (CDR).** Shown for the two main 2°C scenarios that include CDR.

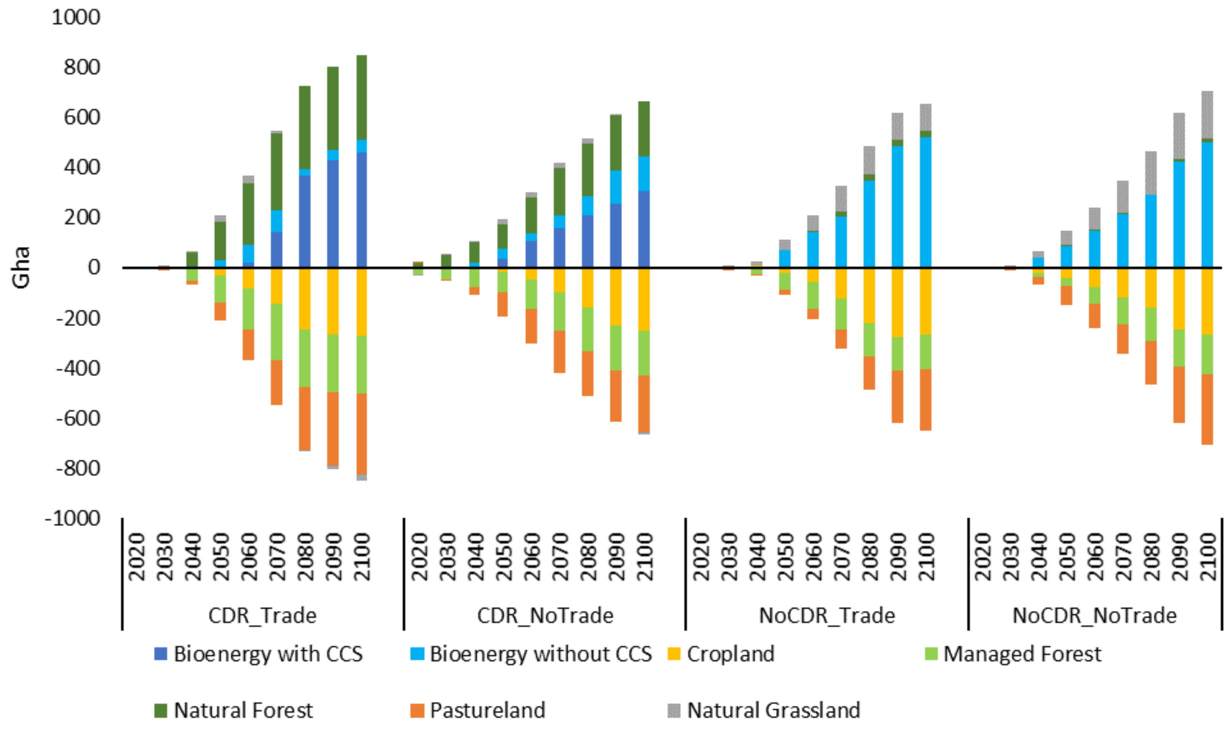

Supplementary Figure 5. Global changes in major land use categories. Shown relative to 2020 in the four main 2°C scenarios.

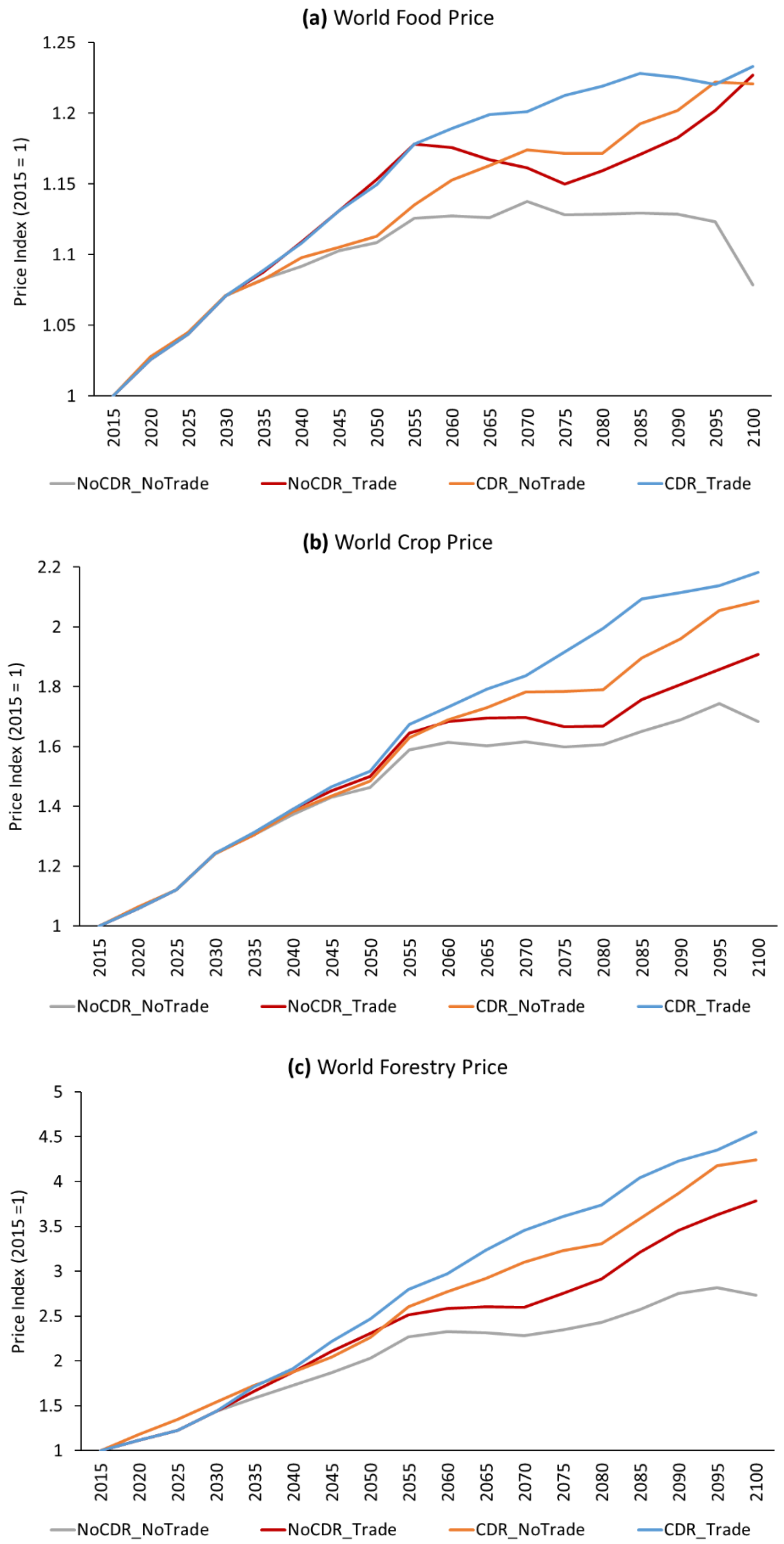

Supplementary Figure 6. **Price indices. (a)** food, **(b)** crops, and **(c)** forestry in the four main 2°C scenarios (2015 =1; note different axes of panels).

2. Sensitivity Analysis

2.1 Considering A/R and BECCS separately

We considered additional 2°C scenarios in which A/R and BECCS were varied separately instead of together. **Supplementary Table 1** displays this larger set of scenarios. The first two and last two rows are identical to the scenarios in the main text. **Supplementary Fig. 7** shows global GHG emissions by type for the full set of scenarios. **Supplementary Fig. 8** compares fossil CO_2 emissions between the CDR_Trade and BECCSonly_Trade scenarios to highlight the difference if A/R does not receive credit for carbon removal, for example due to concerns about the permanence of its sequestration. **Supplementary Fig. 9** shows the A/R and BECCS trajectories with the additional scenario variations. Trajectories are similar when only A/R (or only BECCS) is available compared to when both A/R and BECCS are available, suggesting that the roles for A/R and BECCS are robust to assumptions about the other. **Supplementary Fig 10** shows the overall finding that emissions trading and CDR deployment are mutually reinforcing is robust to the assumption about the treatment of A/R.

Supplementary Table 1. **Extended scenario set for 2°C stabilization target.**

Scenario Name	A/R Emissions Covered under Policy	BECCS Available	International Emissions Trading
NoCDR_Trade			Yes
NoCDR_NoTrade			
A/Ronly_Trade	Yes		Yes
A/Ronly_NoTrade	Yes		
BECCSonly_Trade		Yes	Yes
BECCSonly_NoTrade		Yes	
CDR_Trade	Yes	Yes	Yes
CDR_NoTrade	Yes	Yes	

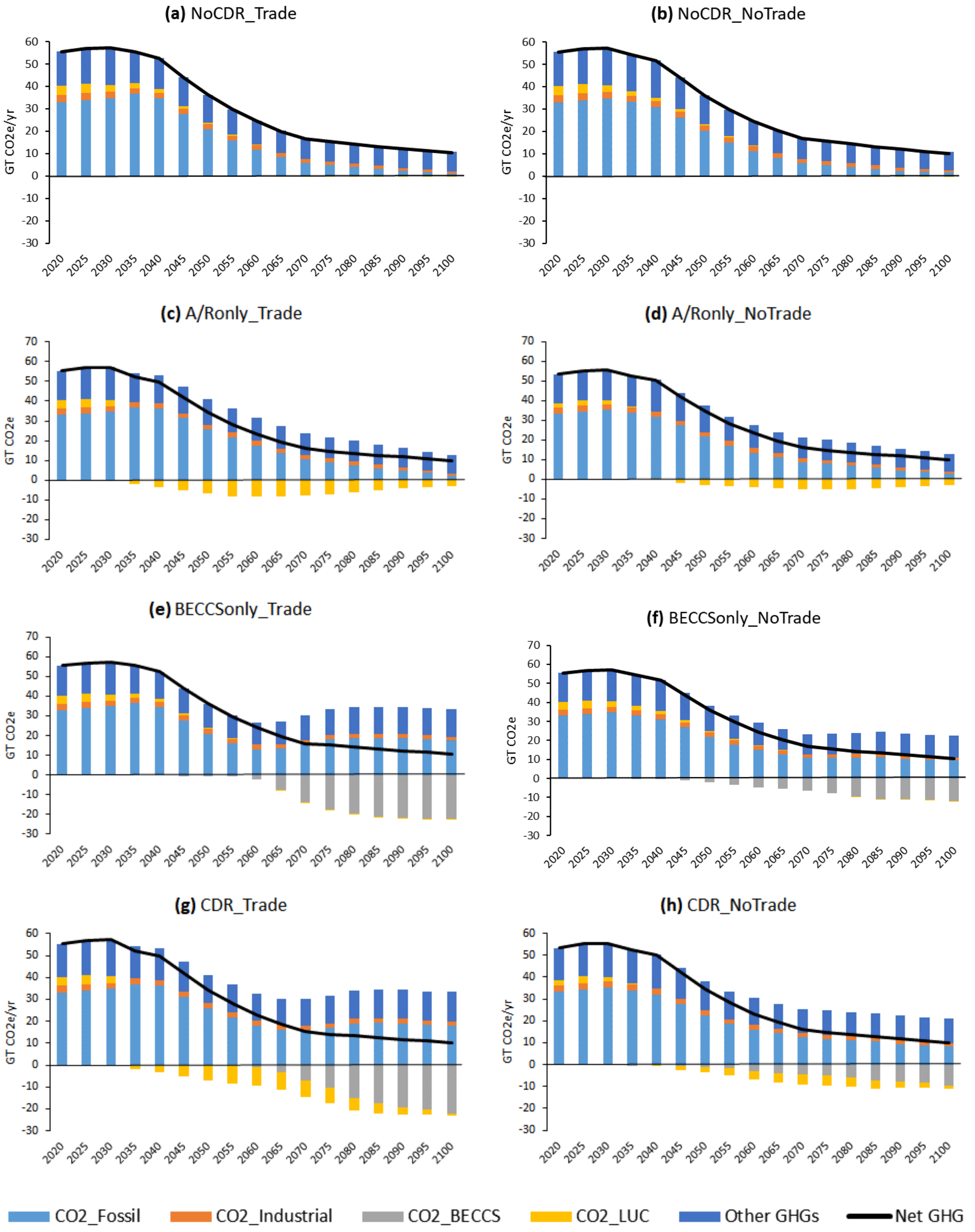

Supplementary Figure7. Global greenhouse gas (GHG) emissions by type in the extended 2°C scenario set. (a) NoCDR_Trade, **(b)** NoCDR_NoTrade, **(c)** A/ROnly_Trade, **(d)** A/ROnly_NoTrade, **(e)** BECCSOnly_Trade, **(f)** BECCSOnly_NoTrade, **(g)** CDR_Trade, and **(h)** CDR_NoTrade. Note: panels (a), (b), (g) and (h) are the same as the panels in Supplementary Figure 3.

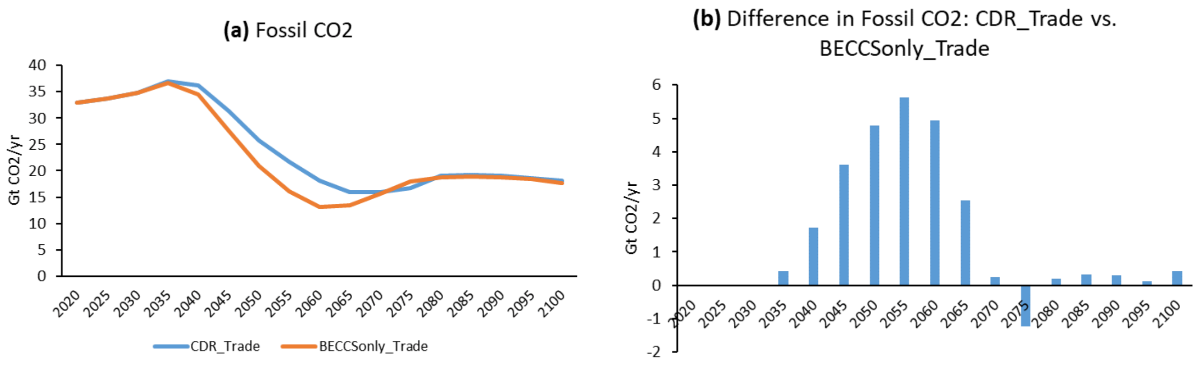

Supplementary Figure 8. Fossil CO2 emissions in CDR_Trade and BECCSonly_Trade scenarios. (a) Fossil CO2 emissions in CDR_Trade and BECCSOnly_Trade over time, **(b)** Difference in fossil CO2 emissions between the CDR_Trade and BECCSOnly_Trade scenarios.

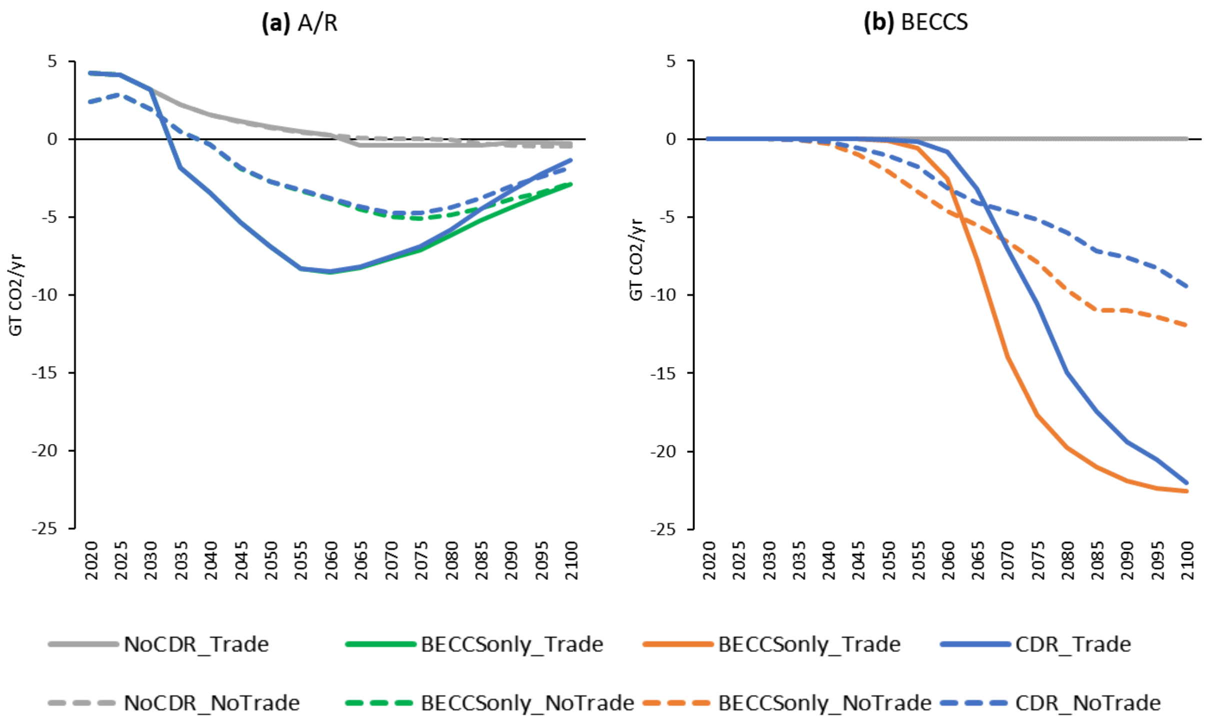

Supplementary Figure 9. Carbon Dioxide Removal (CDR) by type in the extended set of 2°C scenarios. (a) Afforestation/Reforestation (A/R) and **(b)** bioenergy with carbon capture and storage (BECCS) /R and **(b)** BECCS.

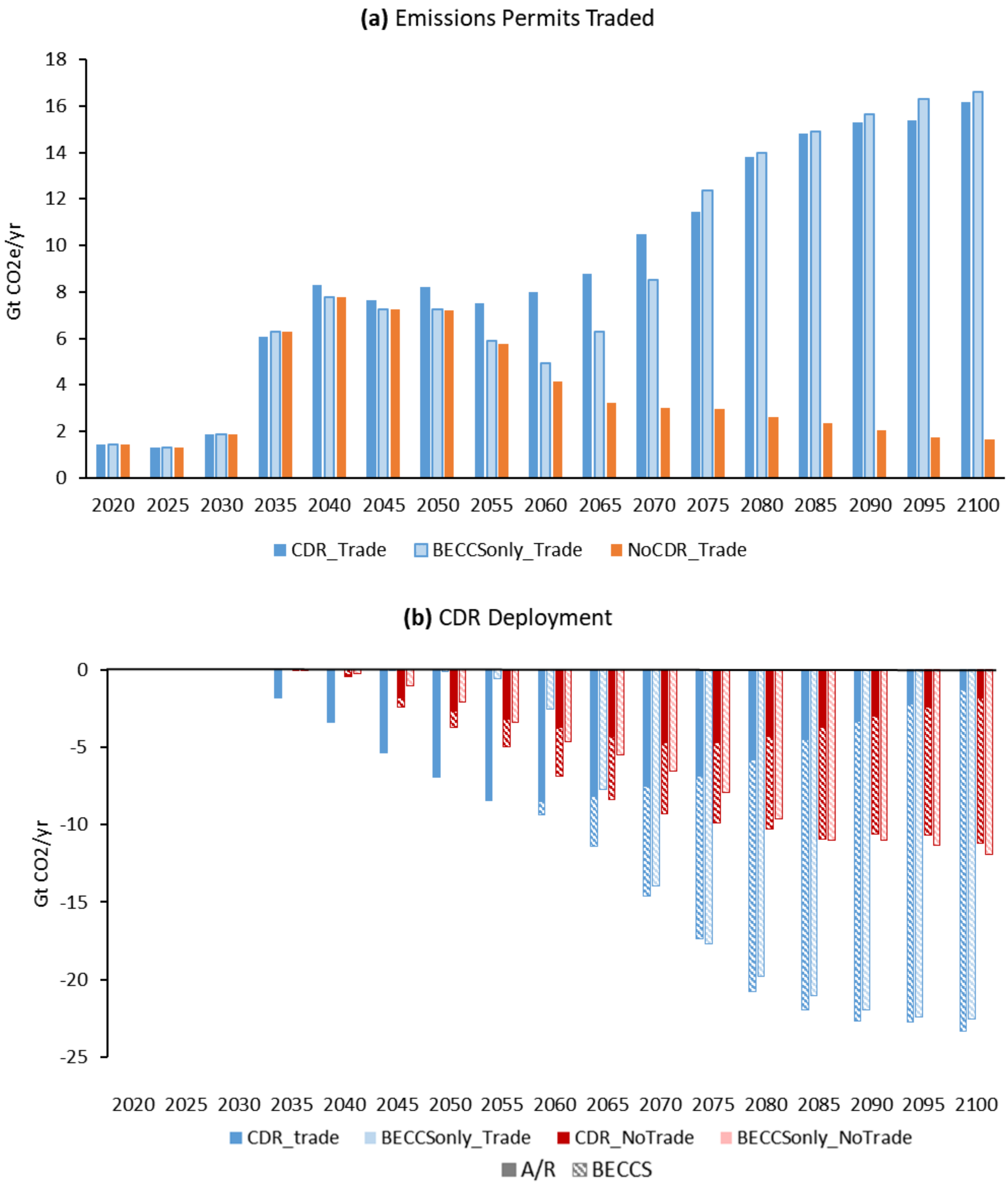

Supplementary Figure 10. The Relationship between Land-based Carbon Dioxide Removal and Emissions Trading in BECCSonly 2°C scenarios. (a) Greenhouse gas (GHG) emissions permits traded globally with both afforestation/reforestation (A/R) and bioenergy with carbon capture and storage (BECCS) vs. with just BECCS (no A/R) vs. with no CDR. **(b)** CDR Deployment globally with vs. without international emissions permit trading with both A/R and BECCS vs. with just BECCS (no A/R). A/R in panel (b) is defined as net negative land CO_2 emissions (net positive land use emissions in early years are not shown) and BECCS removals (land use change emissions excluded).

2.2 Alternative assumptions for BECCS and A/R

We also considered a set of sensitivity scenarios that assume the same 2°C stabilization target as in the main scenarios, but with more favorable assumptions for either BECCS or A/R. For BECCS, we assume no constraints on the deployment rate of BECCS. In the EPPA model, this means removing the technology specific factor (TSF) which typically limits the initial expansion of a new technology by adding costs to early deployment (Morris et al., 2019b). In the absence of the TSF constraint (No_TSF case), BECCS can deploy earlier and faster. For A/R, we assume no limits on where A/R can deploy. Typically, we assume afforestation is limited in each region by the amount of area that is ecologically suitable to grow forests (Griscom et al., 2017). Without that constraint (NoAffLim case), more A/R can be deployed. **Supplementary Fig. 11** shows the A/R and BECCS trajectories with these additional sensitivity cases. Assumptions that favor one of these CDR options tend to have only a modest impact on the other, suggesting the roles for both A/R and BECCS are robust to assumptions about their deployment rate or potential.

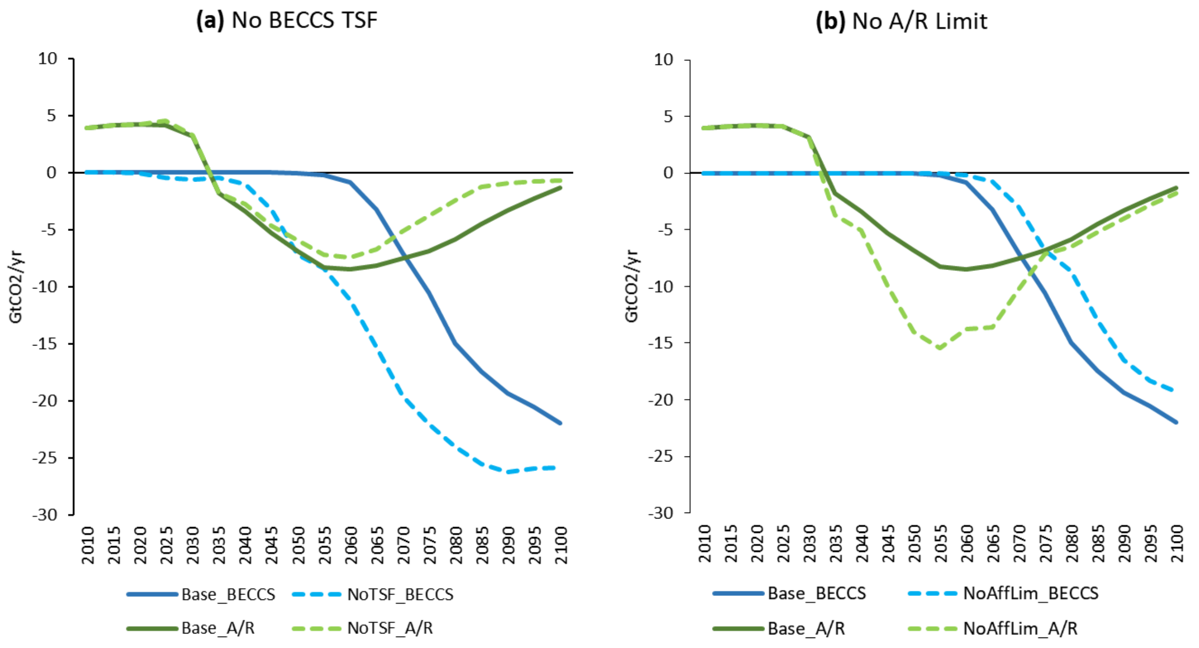

Supplementary Figure 11. Afforestation/Reforestation (A/R) and Bioenergy with Carbon Capture and Storage (BECCS) under alternative assumptions. (a) Alternative assumptions are more favorable BECCS (no technology specific factor (TSF) constraint to how quickly BECCS can expand), and **(b)** Alternative assumptions are more favorable A/R (not limit to A/R). 'Base' refers to the main 2°C CDR_Trade scenario.

2.3 Near 1.5°C Scenarios

We also consider scenarios reaching temperature levels below 2°C, but slightly above 1.5°C (1.6°C) with similar assumptions regarding CDR and trade. **Supplementary Figs. 12-S14** show the volume of GHG emissions traded globally, emissions and permits provided by BECCS and A/R under alternative trade

regimes, carbon prices, total spending on international offsets, and total revenue received by CDR producers in the near 1.5°C Scenarios.

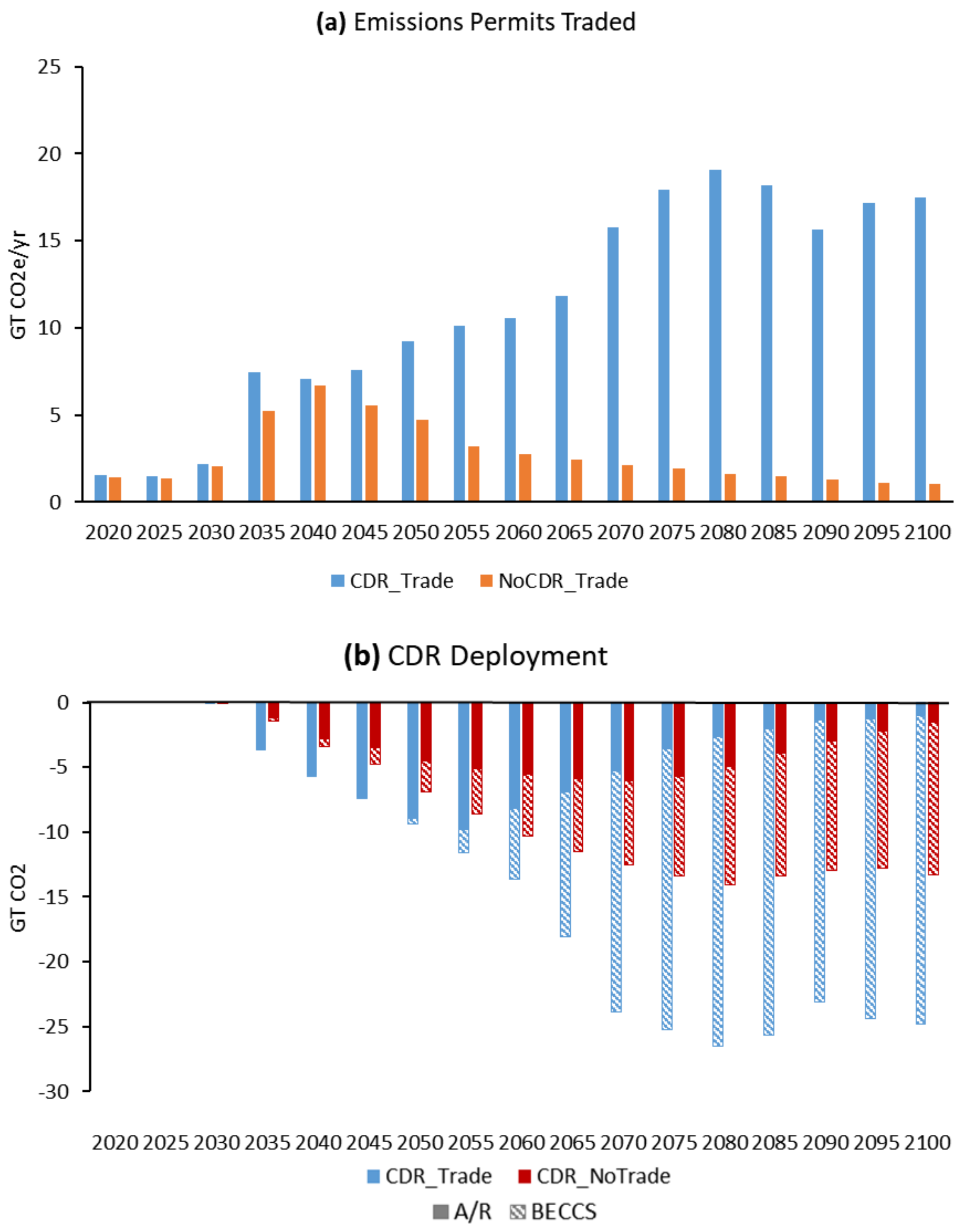

Supplementary Figure 12. The Relationship between Land-based Carbon Dioxide Removal (CDR) and Emissions Trading in 1.5°C scenarios. (a) Greenhouse gas (GHG) emissions permits traded globally with vs. without Carbon Dioxide Removal (CDR); **(b)** CDR Deployment (Afforestation/Deforestation (A/R) and

Bioenergy with carbon capture and storage (BECCS)) globally with vs. without international emissions permit trading. A/R in panel (b) is defined as net negative land CO_2 emissions (net positive land use emissions in early years are not shown) and BECCS removals (land use change emissions excluded).

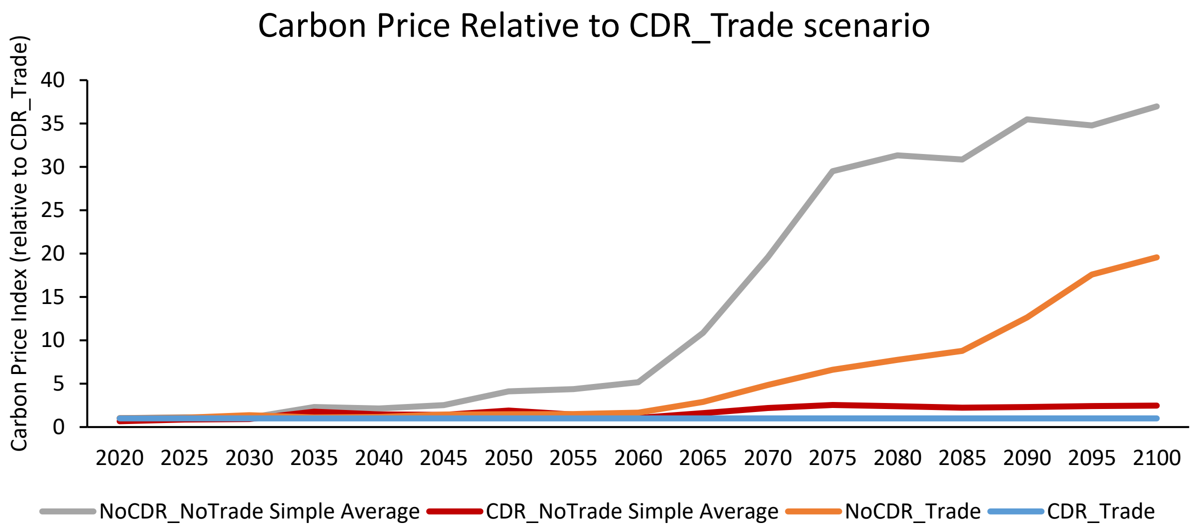

Supplementary Figure 13. Carbon price index relative to the CDR_Trade scenario in the near 1.5°C scenario. For the scenarios without international trading, the carbon prices are reported as weighted averages of the regional carbon prices weighted by the regional cap at time t.

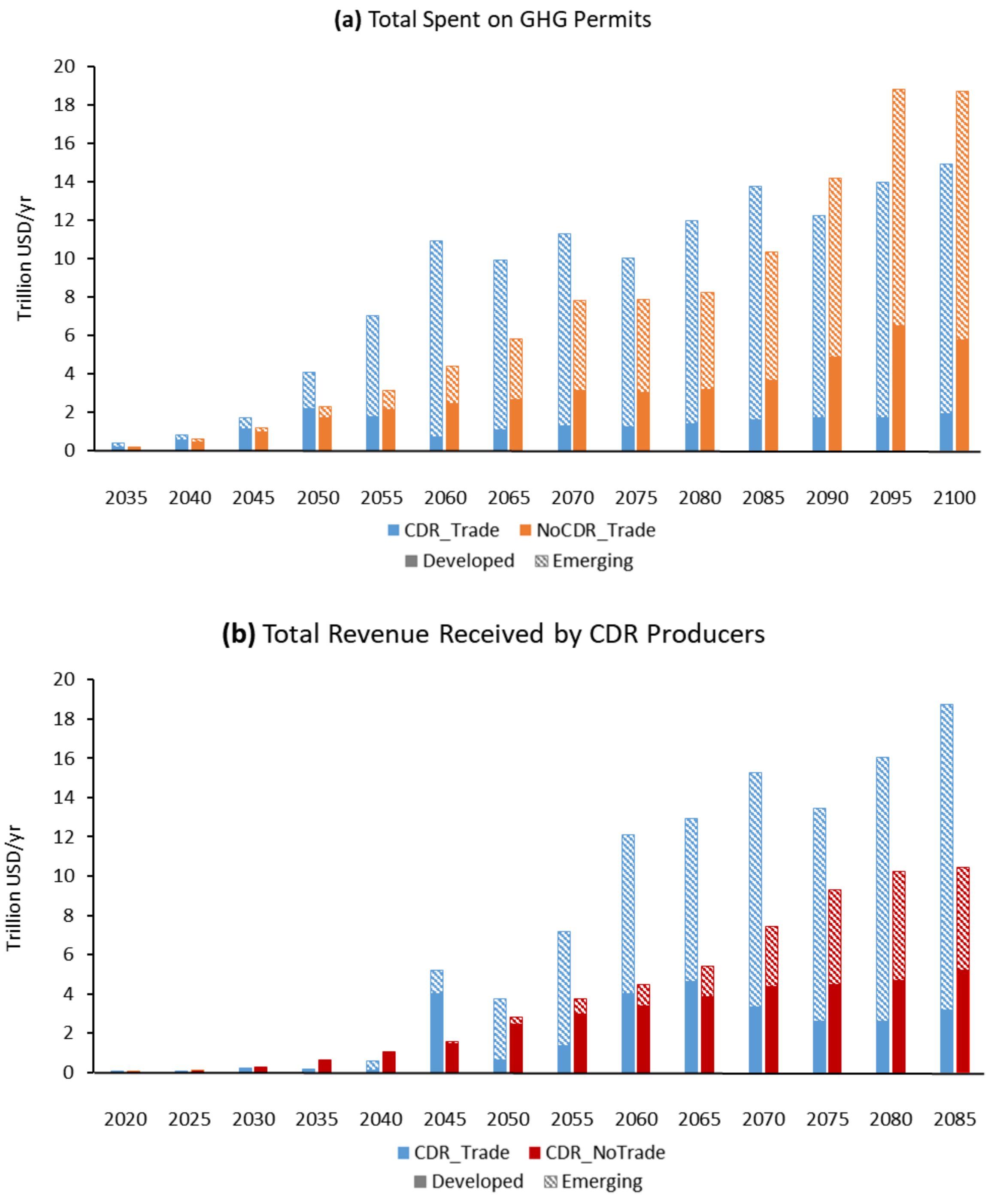

Supplementary Figure 14. Transfers in the 1.5°C scenarios. **(a)** Total amount spent on greenhouse gas (GHG) permits, and **(b)** Total revenue received by carbon dioxide removal (CDR) producers. Dollar values are real 2007 USD. See Supplementary Fig. 1 for definitions of Developed and Emerging regional groupings.

__2.4 Higher emissions cap (less stringent policy) for China:__

We also explore an alternative version of the CDR_Trade scenario in which we increased the emissions cap for China, leading to a less stringent policy in that country (**Supplementary Fig. 15a**). We did not adjust the global emissions, so this test results in higher global emissions and is no longer a 2°C with 66% likelihood scenario, but the deviation in global temperature would be small relative to the main 2°C scenario (**Supplementary Fig. 15b**). With this higher cap, China is a seller of emissions permits around mid-century (when other countries have stringent targets and BECCS has yet to deploy at scale), but remains a buyer of emissions in the longer term as the cost of offsets from BECCS becomes cheaper than abatement options in China (**Supplementary Fig. 16**).

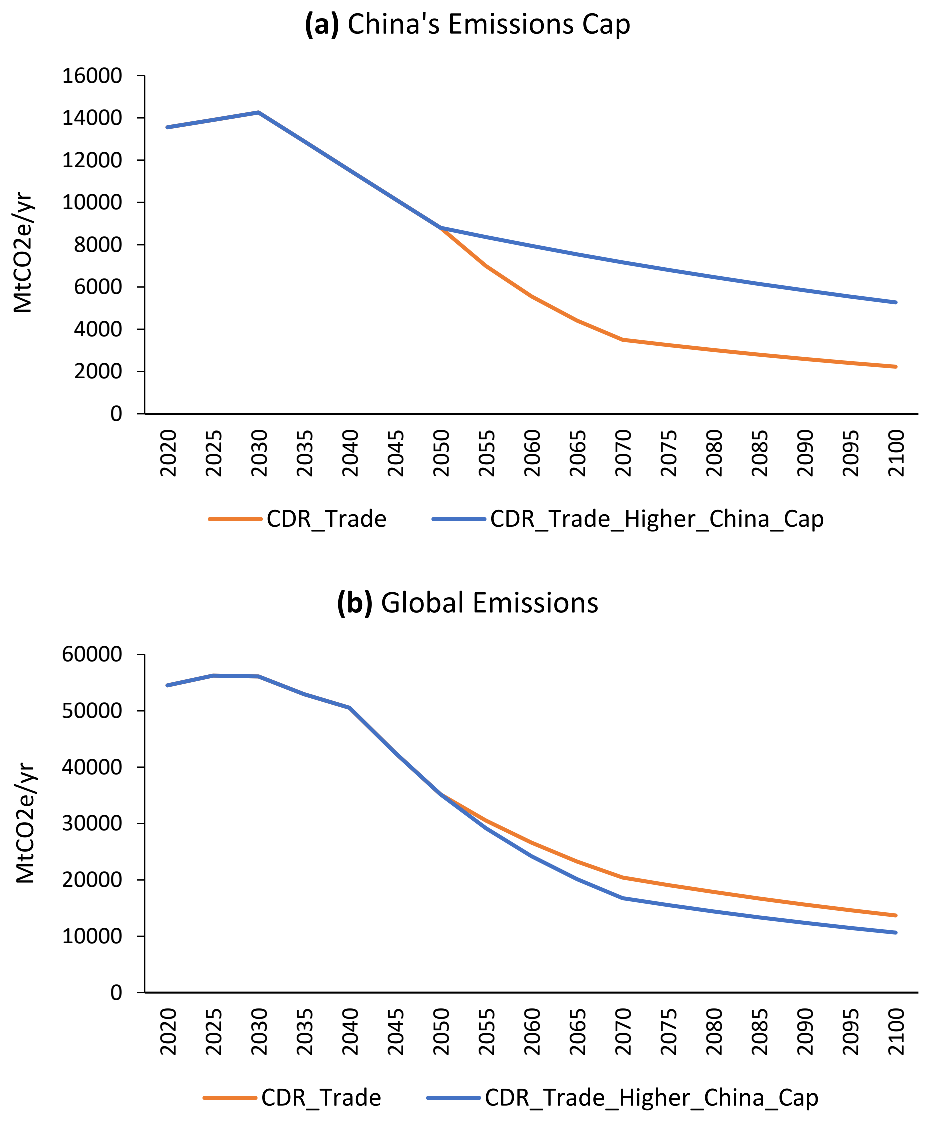

Supplementary Figure 15. China and Global Greenhouse gas (GHG) emissions pathways. (a) China's emissions cap in the main CDR_Trade scenario vs. an alternative CDR_Trade scenario with a higher emissions cap for China, and **(b)** global GHG emissions in the main CDR_Trade scenario vs. an alternative CDR_Trade scenario with a higher emissions cap for China.

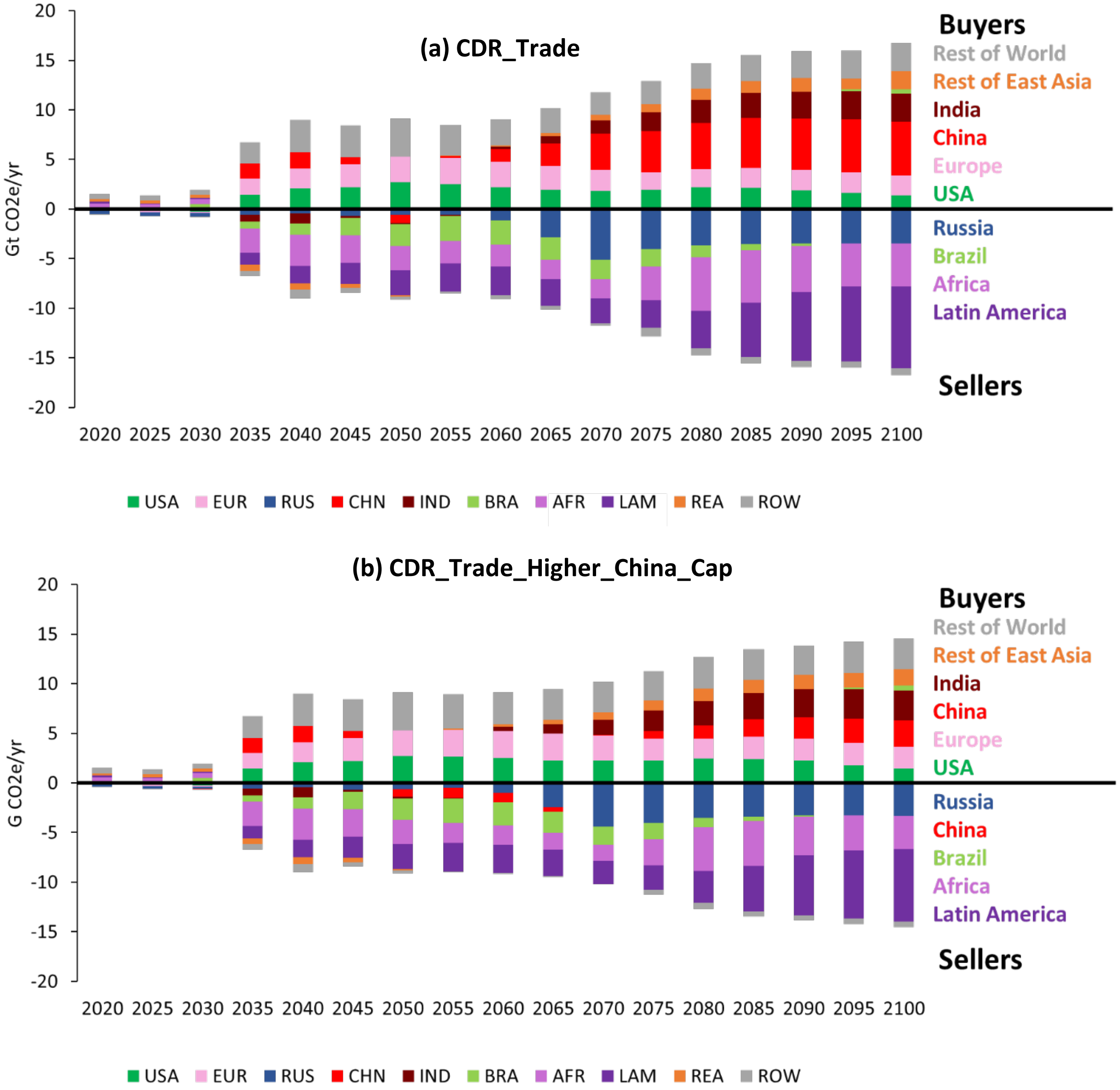

Supplementary Figure 16. Greenhouse gas (GHG) permits bought (positive values) and sold (negative values) by region in 2°C scenarios allowing trade. For a given region, permits bought is calculated as emissions minus emissions cap. **(a)** CDR_Trade case with original emissions caps (same as Figure 3a in Main text) and **(b)** CDR_Trade with higher emissions cap for China. USA = United States; EUR = Europe (EU+); RUS = Russia; CHN = China; IND = India; BRA = Brazil; AFR = Africa; LAM = Other Latin America; REA = Other East Asia; ROW = Rest of World. Regional definitions are provided in Fig. S1 of the Supplementary Material.
